# Using REDCap to track stakeholder engagement: A time-saving tool for PCORI-funded studies

**DOI:** 10.1017/cts.2019.444

**Published:** 2020-02-06

**Authors:** Sabina B. Gesell, Jacqueline R. Halladay, Laurie H. Mettam, Mysha E. Sissine, B. Lynette Staplefoote-Boynton, Pamela W. Duncan

**Affiliations:** 1 Department of Social Sciences and Health Policy and Department of Implementation Science, Wake Forest School of Medicine, Winston-Salem, NC, USA; 2 Department of Family Medicine, University of North Carolina School of Medicine at Chapel Hill, Chapel Hill, NC, USA; 3 Department of Epidemiology, Gillings School of Global Public Health, University of North Carolina at Chapel Hill, Chapel Hill, NC, USA; 4 Department of Neurology, Wake Forest School of Medicine, Winston-Salem, NC, USA; 5 Brody School of Medicine, East Carolina University, Greenville, NC, USA

**Keywords:** Community-based participatory research, research design, comparative effectiveness research, patient engagement, pragmatic clinical trial, stroke

## Abstract

**Background:**

Research Electronic Data Capture (REDCap) is a secure, web-based electronic data capture application for building and managing surveys and databases. It can also be used for study management, data transfer, and data export into a variety of statistical programs. REDcap was developed and supported by the National Center for Advancing Translational Sciences Program and is used in over 3700 institutions worldwide. It can also be used to track and measure stakeholder engagement, an integral element of research funded by the Patient-Centered Outcomes Research Institute (PCORI). Continuously and accurately tracking and reporting on stakeholder engagement activities throughout the life of a PCORI-funded trial can be challenging, particularly in complex trials with multiple types of engagement.

**Methods:**

In this paper, we show our approach for collecting and capturing stakeholder engagement activities using a shareable REDCap tool in one of the PCORI’s first large pragmatic clinical trials (the Comprehensive Post-Acute Stroke Services) to inform other investigators planning cluster-randomized pragmatic trials. Benefits and challenges are highlighted for researchers seeking to consistently monitor and measure stakeholder engagement.

**Conclusions:**

We describe how REDCap can provide a time-saving approach to capturing how stakeholders engage in a PCORI-funded study and reporting how stakeholders influenced the study in progress reports back to PCORI.

## Introduction

Concerned with the lack of inclusiveness of relevant stakeholders in the research process and the excessive amount of time it often takes for effective interventions to be adopted into routine care, the Patient-Centered Outcomes Research Institute (PCORI) has become one of the most influential promoters of stakeholder-engaged research by institutionalizing the involvement of non-traditional research partners in the projects it funds [1]. One of the PCORI’s goals is to advance the science of engagement by understanding the impact of engagement activities on research processes and outcomes.

As a relatively new domain of research, the stakeholder engagement literature is limited but growing [2–4]. The hope is that stakeholder engagement can enhance the relevance of research, increase transparency as to how it is performed, and speed the rate at which research findings are translated into practice [5, 6]. A key aspect of advancing the science of engagement is to understand how best to capture the views and contributions of stakeholders, both to include these data in analyses and to better understand how stakeholders influence research design, implementation, and dissemination activities and venues.

The purpose of this paper is to share our experience with developing and enhancing a method for capturing stakeholder engagement activities using a shareable Research Electronic Data Capture (REDCap) tool. This tool was developed specifically for tracking stakeholder engagement in one of the PCORI’s first large-scale, multi-center pragmatic clinical trials, the 5-year Comprehensive Post-Acute Stroke Services (COMPASS) study [7]. In the COMPASS study, we compared the effectiveness of a comprehensive post-acute care model versus usual care for patients with mild to moderate stroke or transient ischemic attack (TIA) in 40 North Carolina (NC) hospitals. In this paper, we focus on how the Stakeholder Engagement Tracker (SE Tracker) tool was created, enhanced, and used in the COMPASS study. In a future paper, we will describe how stakeholders influenced the study.

## Methods

Between 2016 and 2018, approximately 6000 stroke and TIA patients participated in Phase 1 and 4000 in Phase 2 of the COMPASS trial [7]. The COMPASS research team was large, multi-disciplinary, and geographically dispersed. Community partners were located in rural and urban areas of western, central, and eastern NC, and academic researchers were based out of five universities across NC.

Institutional review board (IRB) approval was granted through Wake Forest University Health Sciences (central IRB) or through local hospital IRBs. COMPASS was granted a waiver of consent and Health Insurance Portability and Accountability Act authorization; therefore, eligible patients were enrolled at hospital discharge. Ninety days post-discharge, patients or their proxies provided verbal informed consent via telephone when self-reported outcomes were collected [8]. COMPASS was periodically reviewed by an independent data and safety monitoring board.

The COMPASS team understood the value of including the voices and suggestions of patients and providers, and many of them had experience engaging community partners in research. However, no study team member had before implemented a pragmatic trial that aimed to change systems of care in multiple health systems in partnership with diverse stakeholders. To capture diverse perspectives and institutions in the trial design and implementation, we included many stakeholder groups (patients, family caregivers, clinicians, hospitals, community-based social service providers, and policy makers) who leveraged their professional and social networks to continually engage stakeholders throughout the trial [9]. This approach shared the workload and put to best use the credibility of team members with specific stakeholder groups. For example, our patient advocate communicated effectively with stroke patients and caregivers, our public health nurses collaborated with community-based social service organizations, and our stroke clinical team members spoke effectively with other clinical teams. Thus, the study team used a shared leadership model so that multiple investigators led engagement activities throughout the study. Additionally, key representatives from each of the above stakeholder groups actively participated with the research team in weekly Steering Committee meetings which helped guide the overall study and allowed for continuous input into decision making. The COMPASS team planned on continually and systematically capturing engagement activities, in part to comply with PCORI’s reporting requirements. PCORI provided guidance as to what was expected in the stakeholder engagement section of their 6-month progress reports. To capture the required data, we developed a stakeholder engagement tracking tool. We chose to build the COMPASS SE Tracker in REDCap [10] due to our familiarity and access to REDCap resources via affiliations with universities that held Clinical and Translational Science Awards (CTSA).

To organize the tool, we first reviewed the PCORI Progress Report template for guidance. Our initial prototype (Version 1) was created in August 2015. It was tested by team member stakeholders and revised accordingly to include 57 questions (fields) in its final version. Our tracking system was designed to capture (1) all engagement activities throughout all phases of the trial, (2) progress along our engagement processes for individual activities, (3) information for PCORI Progress Reports in a format that reduced investigator burden during report writing, and (4) how engagement activities impacted study decisions and processes.

In Version 1 of the SE Tracker, we tested different methods of data collection. We felt that this was important because multiple team members were interacting with multiple stakeholder groups through diverse venues. By offering options for data collection, we sought to minimize the burden of data entry and increase data capture rates by our Steering Committee members described previously.

First, we asked each team member who engaged stakeholders to log into the SE Tracker and document their engagement activities. However, since this was a new research activity for many, team members often failed to directly access the data collection tool. We then tried a different approach and created a survey with a small subset of questions and emailed it to Steering Committee members after they had reported out on their stakeholder engagement work during weekly Steering Committee meetings, as a reminder to document their engagement activities. However, return rates still lagged. This prompted the final change in our process: one investigator (LM) was charged with keeping the SE Tracker current. During weekly Steering Committee meetings, she documented recent stakeholder engagement activities discussed and followed up with investigators individually via email or phone to add further data to the SE Tracker. Version 1 of the SE Tracker includes 285 records (August 2015–June 2017).

We created a simplified SE Tracker Version 2 to reduce study team members’ data entry to the most essential variables (see Table 1). This version reflected suggested changes from COMPASS Team members most actively involved in stakeholder engagement. Version 2 included skip patterns to reduce data entry burden and 39 total possible questions (fields). For example, when a stakeholder and investigator co-presented information about the study to the community, there were 12 items, rather than 57 items, to complete (79% decrease in data entry burden in this case). We streamlined our engagement processes as well. For example, we removed a requirement to have stakeholder input vetted by the overall COMPASS executive committee, reduced the number of types of engagement activities to choose from, and removed a required due date for following up on every stakeholder activity. In Version 2, we also added a feature to allow for uploads of documents in PDF form, rather than entering all information directly into REDCap. In the interest of time, we may have provided only brief open-ended responses for stakeholder data forms. However, we found alternatives, such as uploading documents that still captured rich details about the engagement activities. For example, one PDF is the multi-paged letter we sent to focus on group participants summarizing the themes they raised and how we would address them in the design of the intervention or training of providers delivering the intervention. We accessed this PDF during the writing of our Final Progress Report to PCORI and during the writing of a manuscript on how stakeholders influenced the study.

**Table 1.** Comparison of Versions 1 and 2 of the Stakeholder Engagement Tracker

Information collected	Version 1	Version 2	Reason for change
Engagement activity general information	✓	✓	
Method/type of stakeholder engagement	✓	✓	
Skip patterns on type of engagement	☒	✓	Reduce data entry
Open-ended questions on challenges and successes	✓	✓	
Stakeholder engagement activity request	✓	☒	Study team entered all data
Stakeholder engagement activity impact on study	✓	✓	
Reason for stakeholder input not being included or implemented	✓	☒	n/a; stakeholder input required field in Version 2
Phase of study	✓	✓	
PCORI level of engagement	✓	✓	
Stakeholder follow-up notes	✓	☒	Used drop-down box to reduce open text
Document uploading	☒	✓	Supporting documentation option reduced data entry

Over a 3-day period (∼13 hours), a team member built, tested, and revised Version 2 of the SE Tracker. It was put into production at the end of June 2017 after a progress report was sent to PCORI using Version1 of the SE Tracker, a meaningful breaking point. As of October 1, 2019, a total of 208 stakeholder engagement records were created; data entry took on average ∼6 minutes per entry. We spent an another 40 hours developing Version 2 of the SE Tracker database, and designing reports that we then re-ran for future progress reports to PCORI.

Version 2 of the SE Tracker was used throughout the duration of COMPASS study, following the Study Design Phase. Version 2 still met all PCORI reporting requirements but captured less detail and took less time to manage. For example, Version 1 included a checkbox for potential duplicates. COMPASS team members and various stakeholders were entering data directly or via the survey tool and unable to determine potential duplication. However, this was removed in Version 2 since one person was doing the data entry and could review the records with similar titles or subjects to reduce duplication. The data dictionary has been frequently requested by other PCORI-funded study teams and CTSA’s Community Engagement Programs and is shared here for others to tailor to their needs (see Online Supplement 1 for the Excel data dictionary; see Online Supplement 2 for PDF of REDCap form).

### Data Fields in the REDCap SE Tracker

**Fig. 1.**
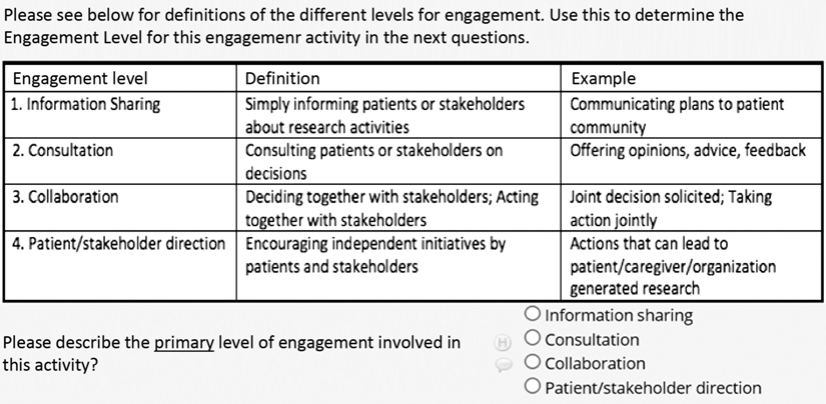
PCORI levels of stakeholder engagement in Stakeholder Engagement Tracker using REDCap.

Below we describe the data fields we found most helpful in our SE Tracker Version 2 and would recommend to others for inclusion. Guided by the questions PCORI requested in 6-month progress reports, data capture included which stakeholder groups were involved; when, where, why, and how they were engaged; their level of engagement; and how challenges to engagement were overcome. Some fields were open-ended and others forced-choice. This allowed us to capture rich information and simultaneously compute descriptive statistics about our engagement. None of the fields were prescribed, reviewed, or endorsed by PCORI. Other stakeholder groups were not involved in the creation or refinement of the tool. We used PCORI frameworks whenever available. For example, in describing completed engagement activities each reporting period, we used PCORI’s definition of four levels of engagement (Fig. 1).

**Fig. 2.**
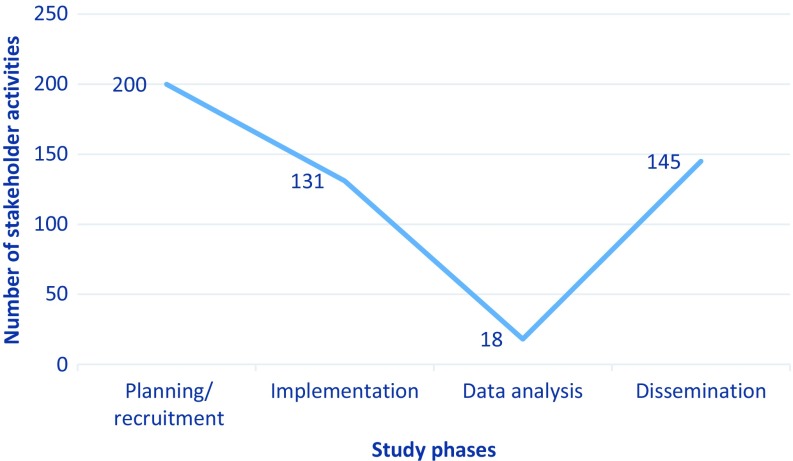
Tracking stakeholder engagement across study phases.

The tool served as an evaluation tool for the Engagement Team and was reviewed monthly. For example, we added forced-choice data fields to monitor stakeholder engagement across the four study phases of planning, implementation, data analysis, and dissemination (Fig. 2). While we strove to be equitable in involvement, fewer opportunities for meaningful involvement arose during the data analysis phase, because it required strict confidentiality and rigorous, specialized methods with which few team members were familiar. Our data prompted us to ask if there were other opportunities for meaningful involvement of people outside the research team to inform data analysis. As a result, we asked the state’s Medicaid administrator how to break out patient demographics so that the data were most useful to such stakeholders.

**Fig. 3.**
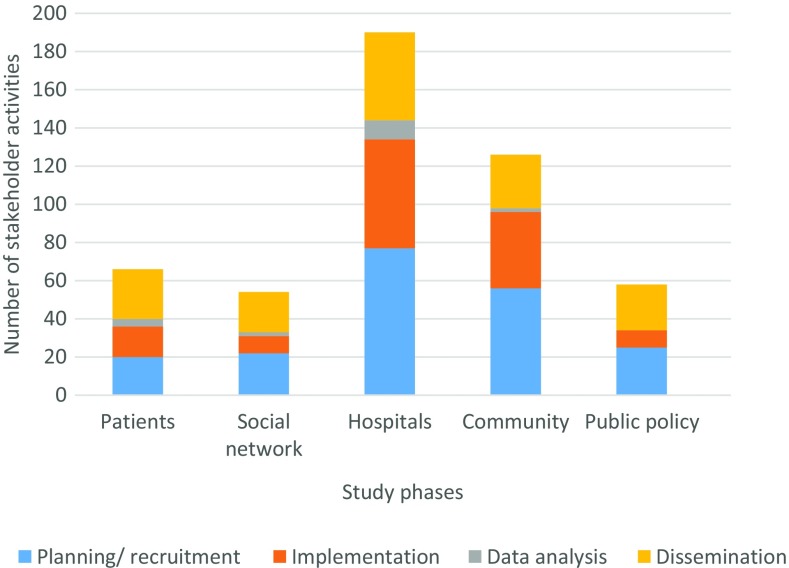
Socioecological model of stakeholder engagement activities across study phases.

To monitor whether we were engaging all concentric circles of influence on patient health according to the socioecological model [11] (individual, social network, institutional, community, and public policy), we added other forced-choice data fields to code stakeholder engagement activities. The goal was to increase our chances of successful systems change – so we monitored where we were putting most of our efforts and whether we were striking a balance of contributions among our various stakeholder groups (Fig. 3). Our data showed us that we were indeed engaging all spheres of influence. The data also highlighted that most engagement activities were occurring at the hospital level, which was reasonable given that that is where the new care model intervention was being delivered.

We added open-ended data fields to capture context, challenges and solutions, influence on study decisions. For example, one entry in the SE Tracker focused on training hospital clinical staff during the Implementation Phase. The challenges included the inability for all relevant staff to attend the training in person, which drove the decision to provide webinar-based training options. Stakeholder feedback (collected via survey after training) also resulted in our shortening trainings from 3 to 2 days and featuring presentations from the clinicians in the first wave of hospitals who had already implemented the intervention in their clinics. These stakeholders focused on how to implement the study under real-world conditions and described how their hospital handled challenges with implementation as they arose. They also provided guidance on which challenges to anticipate and prepare for, to minimize their occurrence at the new sites. We entered into the SE Tracker the feedback clinicians provided, how that changed our training of other clinicians, and then the measured improvement in training event satisfaction ratings from subsequent waves of clinicians we trained.

### Monitoring the Engagement Process

**Fig. 4.**
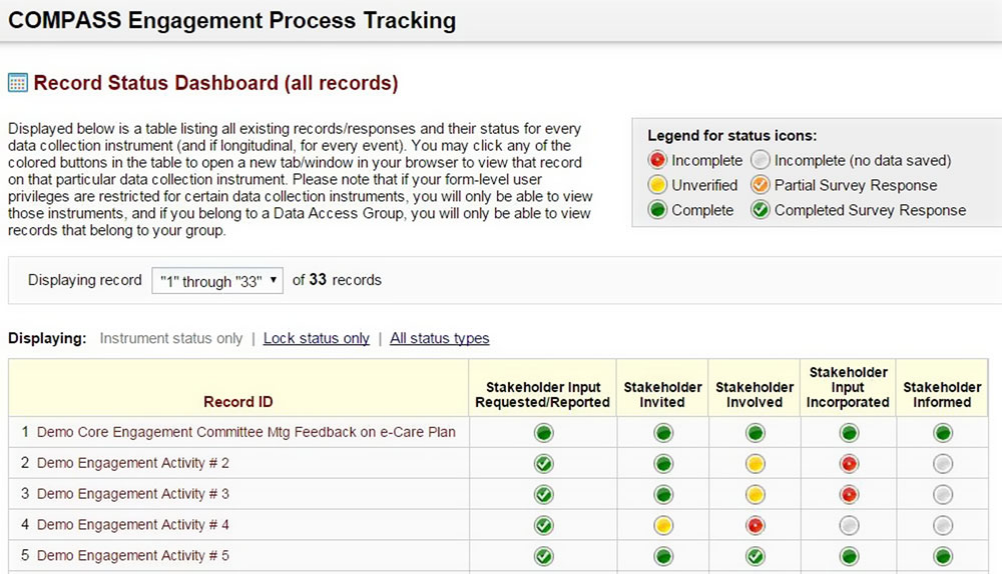
Screenshot from the REDCap Stakeholder Engagement Tracker Dashboard.

We used the Dashboard feature in REDCap to monitor whether each stakeholder engagement activity was completed by following these steps:

- *Invite* stakeholders to participate (identify who our stakeholders are, who will be affected by changes in care delivery, and who can affect changes in care delivery)
- *Include* stakeholders in the research process
- *Incorporate* stakeholder input into study decisions
- *Inform* stakeholders of how we incorporated their input (Fig. 4).

The Dashboard served as a project management tool for the Engagement Team. When the Dashboard demonstrated that we had not completed an engagement cycle, we took action.

## Discussion

Despite calls for improving the quality and context of reporting of stakeholder engagement by developing and validating tools that capture stakeholder engagement in research [5], few tools describe how engagement processes have been supported and implemented [3].

Using REDCap, we created a SE Tracker that served multiple purposes in our PCORI-funded large pragmatic trial. The SE Tracker was our tool for data collection, project management (for engagement activities, not for the study), and evaluation to determine if stakeholders were engaged and made contributions throughout the study. Thus, it was an effective way to document changes to the study informed by stakeholder input. It can also show how different activities are connected across time and influence behavior. For example, the pharmacist on the study team developed a medication reconciliation and adherence toolkit with stroke neurologists and primary care physicians. He then travelled across the state and met with community-based pharmacists who were part of the Community Pharmacy Enhanced Services Network (CPESN^®^ Network), developed by the Community Care of North Carolina (CCNC), the largest and longest-running medical home system in the USA. Together, they developed an implementation strategy including how to consult with local primary care providers and refer COMPASS patients to local participating pharmacies. He discussed COMPASS during a CCNC webinar and encouraged the pharmacy directors from each CCNC region to be resource individuals for local pharmacists who have questions about the medication management intervention in COMPASS. The impact of this effort was a strong participation of pharmacists in the Community Resource Networks established at each participating hospital. These pharmacists then became critical partners to the post-acute care coordinators in caring for patients after discharge with expertise and resources for medication management, smoking cessation, blood pressure management, diabetes management, etc. Finally, we can connect these efforts to the words of one post-acute care coordinator who remembered hearing a local pharmacist’s presentation and reached out to them for assistance on medication management and home delivery to a patient who lived alone, was unable to drive due to the loss of sight, and needed insulin. The pharmacist got the prescription issued from the Veternans Affairs delivered to the patient’s home. The post-acute care coordinator stated that without the COMPASS training on community resources and the new connection to the local pharmacist, the patient would not have the insulin he needed so quickly. She also stated that she had no idea what patients experience after discharge but that the follow-up call opened her eyes. The follow-up call was a component of the COMPASS intervention because a lead patient stakeholder told us “A follow up call is key.” This is the type of powerful story on how stakeholders influenced the study based on data in the SE Tracker.

The SE Tracker can also show stakeholder input was received and used in the short-term. For example, “Did the stakeholders’ involvement with the training of hospital staff receive favorable reviews from participants?,” “Were the stakeholders’ suggestions regarding study materials used in the study?,” “Did the clinical staff find these materials helpful in describing the study to patients and caregivers?”

We found our processes to be exceptionally helpful for completing progress reporting on time and for understanding where and when our stakeholder engagement activities occurred across the state. The literature reports a drop-in engagement in later parts of trials, such as in the analyses, interpretation, and dissemination phases [3, 5, 12]. In Phase 1 of the COMPASS study, we also had a significant drop in engagement activities during the Data Analysis Phase. However, we had high levels of engagement during the Dissemination Phase, during which we actively engaged all stakeholder groups and had a similar number of activities as in the Implementation Phase.

We were unable to find many publications that shared tools for capturing stakeholder engagement activities throughout a project period. While there were papers that made the case for stakeholder research, examples of who to invite as stakeholders and why, and how to plan the engagement processes, details of data capture for these activities were not included [12–14].

One tool (developed by the Department of Communities, Disability Services and Seniors in Queensland, Australia) is an Excel spreadsheet template that can be used and modified as needed (https://www.communities.qld.gov.au/resources/dcdss/disability/ndis/stakeholder-tracking-sheet.xls). This tool is designed to capture contact information about stakeholders, the type of engagement that has occurred, if such engagement was one-on-one versus in small groups versus in larger groups, and a few other items. But because it is not intended to support stakeholder-engaged research, it lacks questions about activities we captured in the SE Tracker (e.g., stage of the project, where the engagement occurred, or if there was a change in process due to the engagement activities). However, this Australian tool is free, easily accessible, and quickly modifiable for others to use.

Other stakeholder engagement resources are used to identify and even prioritize appropriate stakeholders to guide projects and/or serve as templates for planning engagement activities but exclude tools to capture specific data on how and when stakeholder contributions occurred and how they may have changed aspects of the projects. For a sample of these tools, please see: https://www.k4health.org/sites/default/files/migrated_toolkit_files/DDIU_Stakeholder_Engagement2.pdf and https://www.quorum.us/resources/stakeholder-engagement-matrix-template/202/.

### Challenges

The design of the SE Tracker created several challenges. First, to generate accurate reports, we needed to have complete records on all variables (e.g., no duplicate or missing records, all fields completed). Some variables offered clear definitions, such as the level of engagement, but not all. We did not provide training on entering consistent values based on clearly defined categories when it would have been helpful (e.g., whether the Planning Phase of the study was defined as the period of grant writing, ramp up, design of data collection tools, and/or recruitment). A clear study-specific or funder-specific definition of coding variables or a list of activities associated with each study phase could address this issue. PCORI’s current 6-month progress report provides study phases that are straightforward: study question/topic development, design: intervention/tools/comparators, choice of outcomes and measures, study Design: other, participant recruitment/retention, data collection, data analysis or results interpretation, dissemination, and other phase). These would not necessarily require training for consistent data entry.

A second challenge was that we did not build in a systematic method to connect separate records. Currently, the challenges and outcomes are recorded for each activity record. However, another data element that requests linking this record with a related activity would be very useful. To determine how various records were related, after the SE Tracker reports were generated, the Stakeholder Engagement Team reviewed the reports and noted related activities of entries, such as the subject line or name of the activity. However, to determine the progress of stakeholder involvement and the potential challenges and solutions over time for a specific patient stakeholder, all records were reviewed for entries with similar titles referring to that patient. A lack of a linking mechanism increases the likelihood of missing critical information, such as the challenge that this patient stakeholder presented during the planning phase resolved and if so, how it impacted later phases of the study. REDCap does allow for linking of data across the different phases of the study. In Version 1 of the COMPASS SE Tracker, various people entered data via the database or survey tool which did not include a consistent method of naming entries or a way of linking entries. When it became protocol for a single user to enter data, this need to link entries became apparent and more feasible with our workflow. However, by the time it became protocol one user to be responsible for data entry, it was a longer time investment to retrospectively link entries due to the number of entries, than reviewing all entries. We recommend that future teams create a variable with categories in a drop-down menu to link entries across study phases, so study decisions and potential impact can be followed. For example, any entries that related to “Collaboration on consent form” would be linked. These might show that incorporating patient, family members, and IRB feedback during the development of consent form was associated with high rates of consent during implementation, allowing pre-coding of qualitative data during data capture and including a full set of themes for analysis.

### Change that the Tool Does and Does Not Show

With this tool, we cannot demonstrate that stakeholder engagement work directly influenced process metrics such as hospital recruitment, delays in IRB approval, or patient recruitment. However, thanks to the careful design and process of capturing data on stakeholder engagement using the SE Tracker, we can state that the intervention includes certain components (e.g., a phone call from a nurse trained in stroke care 1–2 days after discharge) and materials (e.g., a refrigerator magnet with the nurse’s phone number, instructions for how to talk to patients with aphasia) because patients and their family caregivers explicitly requested them. We know that a consent form written by only researchers was approved by the IRB faster, but then when we included input by patients and family caregivers, it took longer to get IRB approval [8]. The original language about hospitals being randomized to deliver usual care versus the intervention deeply concerned patients and family members, whereas the IRB considered it “the best paragraph in the consent form.” Our stakeholders anticipated that patients informed that they were not receiving the intervention would feel as if they were not receiving optimal care and that would undermine their recovery. In contrast, the researchers and IRB believed the original language clearly stated how randomization was conducted and what patients would experience in either condition. Together, we revised the consent form until we reached consensus. All of this was captured using the SE Tracker so that a more detailed summary on how the various stakeholders influenced the design, implementation, and dissemination of the COMPASS Study could be provided. We did not test whether the final consent form resulted in higher patient recruitment rates but have captured stakeholder influence on the final consent form and how we arrived at a win–win solution.

### Considerations for the Future of Building Stakeholder Tracker Tools

Neither PCORI nor the National Institutes of Health (NIH) has prescribed what information to capture to describe engagement activities, to the best of our knowledge. Standardizing data collection processes could help reduce the variability in quality and content of reporting on stakeholder engagement in research, and advance the science of engagement. Stakeholder engagement researchers might consider using similar strategies for data collection across studies to create more standard methods and augment evidence for the effectiveness of various stakeholder engagement activities. For instance, Concannon et al. [5, 15] grounded their systematic review using the “7 P’s” (patients and the public, providers, purchasers, payers, policy makers, and principal investigators) to categorize relevant stakeholder groups. This method helps to standardize approaches and who to include as stakeholders and allows investigators to see if they have a reasonable balance of stakeholders representing each category. This may help to mitigate the current under-representation of payers, product makers, and purchasers in research [5]. Further standardization could come from including the “7 item questionnaire” for reporting on stakeholder engagement in research also included in the Concannon systematic review. However, we caution against standardization that limits engagement.

Finally, there are likely existing REDCap features that we did not use that would be helpful (e.g., set up weekly automatic emails of the survey to investigators to remind them to document their stakeholder engagement and to complete all fields).

Although this tool was developed to manage a cluster-randomized pragmatic trial, it could certainly be used in other study designs and adopted for different frameworks, due to the tremendous flexibility of REDCap data collection and reporting features. Because our focus was to include stakeholders at all circles of influence on patient health, we tended to organize our data along that framework. However as the dataset is set up now, we could easily report out along levels of engagement (e.g., information sharing, consultation, collaboration, and stakeholder direction) or stages of research (e.g., study design, data collection, data analysis, and dissemination).

### Considerations for CTSA’s Community Engagement Programs

This tool could be adapted and used by CTSA’s Community Engagement Programs to track lessons learned by engaging community stakeholders, input sought and used to improve programs, and whether targets were met and indeed have already been shared with two CTSA’s Community Engagement Programs. Additionally, we shared the tool with a patient stakeholder who is leading another grant effort. The ability to upload the data dictionary from our tracker and then make respective project relevant changes without having to completely recreate a new tool can enhance efficiency.

### Considerations for NIH-Funded Studies

This tool may be adapted to a wider audience than PCORI awardees. By setting up fields to reflect NIH progress reporting requirements, researchers could keep track of the information they upload into the Research Performance Progress Report (RPPR). For example, one might define a set of fields within a section to capture information to report out on “How results have been disseminated to communities of interest?” (RPPR B.5) such as when, where, why, who, how, and lessons learned (“disseminated-when,” “dissemination-where,” etc). Similarly, one might define a single field to capture open-ended text for “Actual challenges or delays and actions taken to resolved them” (RPPR F.2.). The time it takes to track this information during the study would be saved in completing the progress reports.

### Dissemination

Next step for disseminating the tool is to submit it to the REDCap Library Oversight Committee to have it included into the REDCap Shared Library (https://redcapvanderbilt.edu/consortium/library/search.php) which will make this tool available to any REDCap user with the appropriate permissions. There are also opportunities to showcase this tool as a novel use case during weekly REDCap consortium web meetings and or the annual REDCap Consortium Conference which are attended by REDCap administrators worldwide. The tool and this publication will also be featured in our PCORI Final Report which will be posted on the PCORI webpage. Other venues for dissemination include presentations to the CTSA network via the Working Group for Assessment of CTSA Institutions Strategies for Engaging Community Partners, and to the Collaboration and Engagement Domain Task Force.

## Conclusion

Using REDCap, we created a stakeholder engagement tracking tool that served multiple purposes in a large pragmatic trial funded by PCORI. It was a tool for data collection, project management, and evaluation of stakeholder engagement activities. Thus, it was an effective way to meet funder requirements and document changes to the study informed by stakeholder input.

## Supplementary material

For supplementary material accompanying this paper visit http://dx.doi.org/10.1017/cts.2019.444. click here to view supplementary material
