# Supplementary material for: Using REDCap to track stakeholder engagement: A time-saving tool for PCORI-funded studies
Source: J Clin Transl Sci. 2020 Feb 6;4(2):108–14. doi: 10.1017/cts.2019.444 (PMC7159814; doi:10.1017/cts.2019.444)
Supplement: Supplementary file 1 [file S2059866119004448sup.zip › 2059866119004448sup003.pdf]

Importing the COMPASS Stakeholder Engagement Tracker into REDCap

1. Log in to your institution's REDCap
2. If applicable for your institution, select either an Internal or External Project

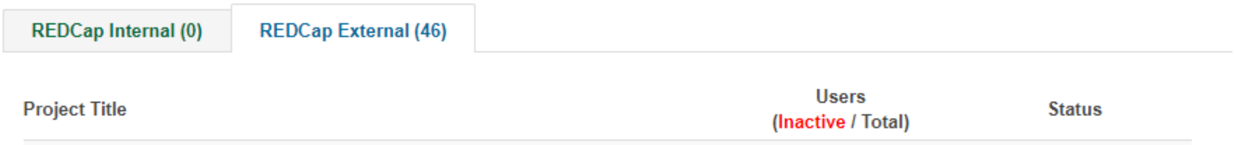

3. Create a new project or open the existing project you would like to add the library
4. If creating a new project, select "Create an empty project (blank slate)"

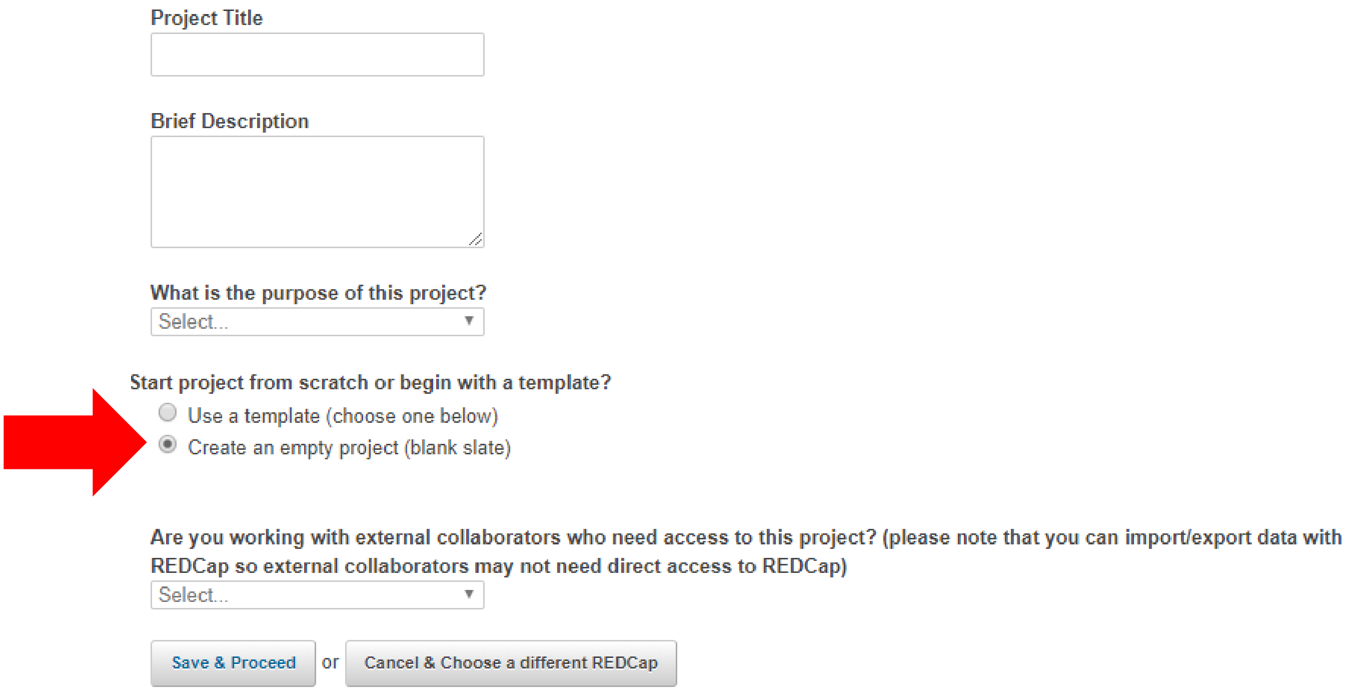

5. Navigate to the “Project Setup” page

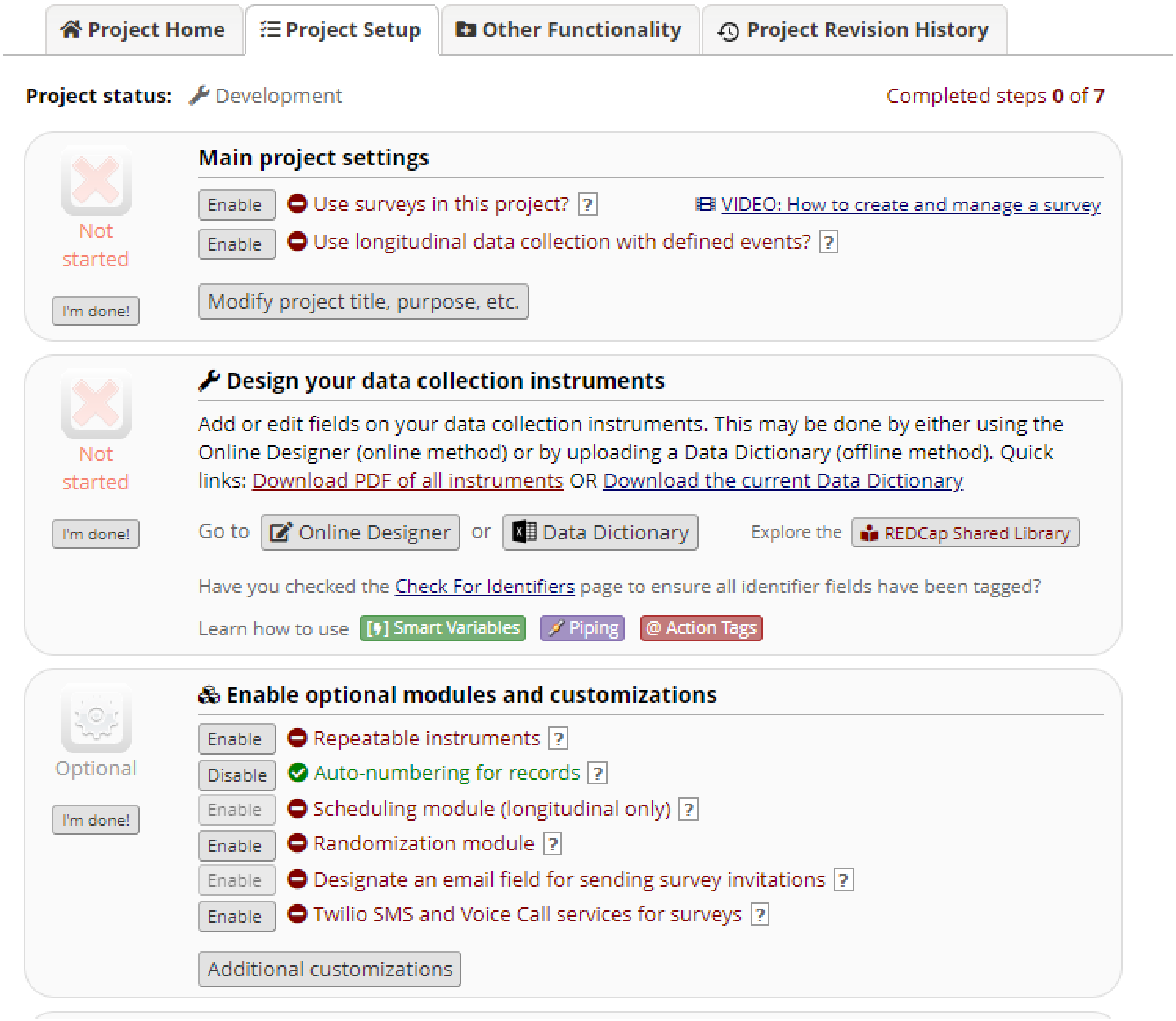

6. Click on the "REDCap Shared Library" button under "Design your data collection instruments"

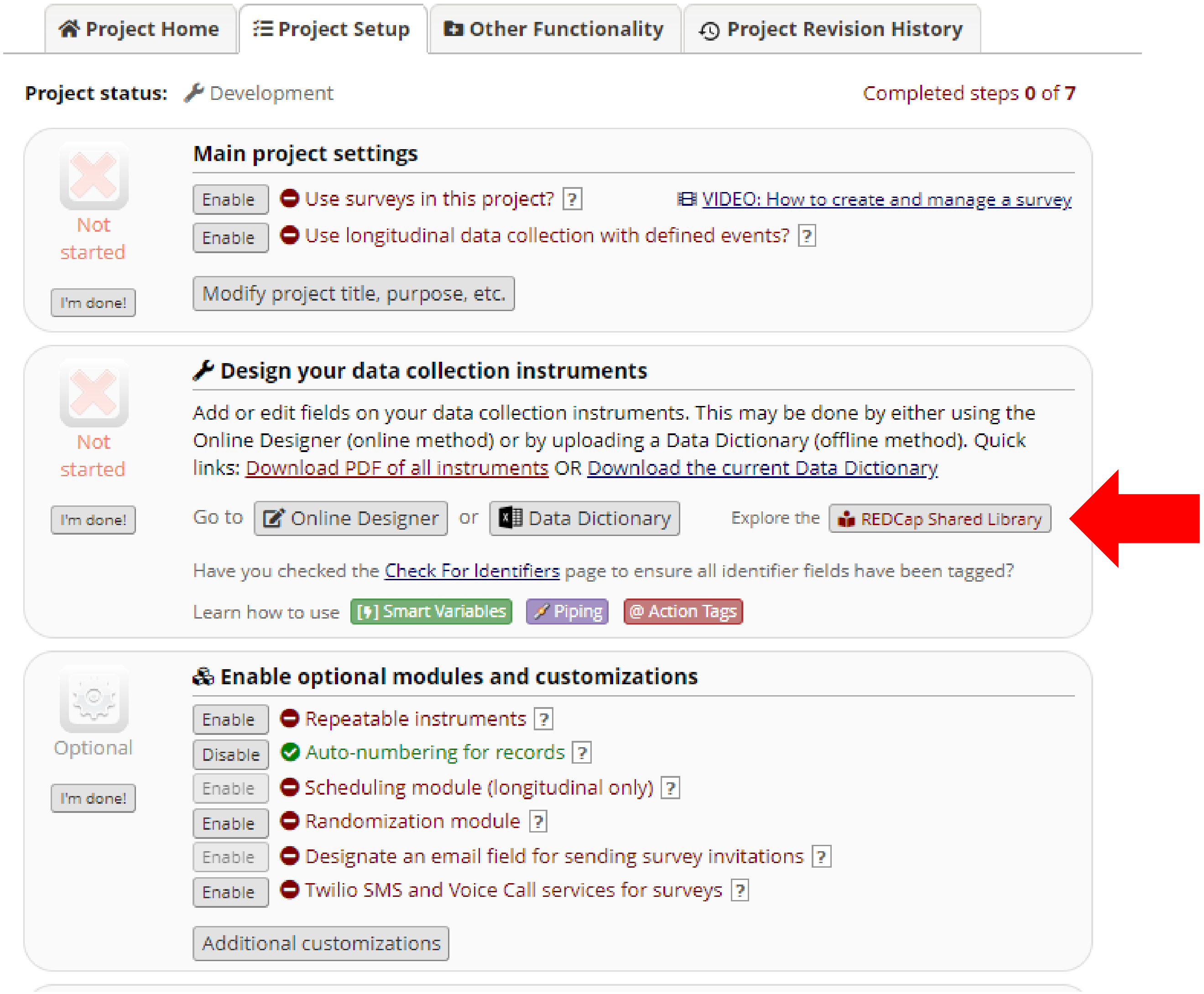

7. You will then be directed to the REDCap Shared Library

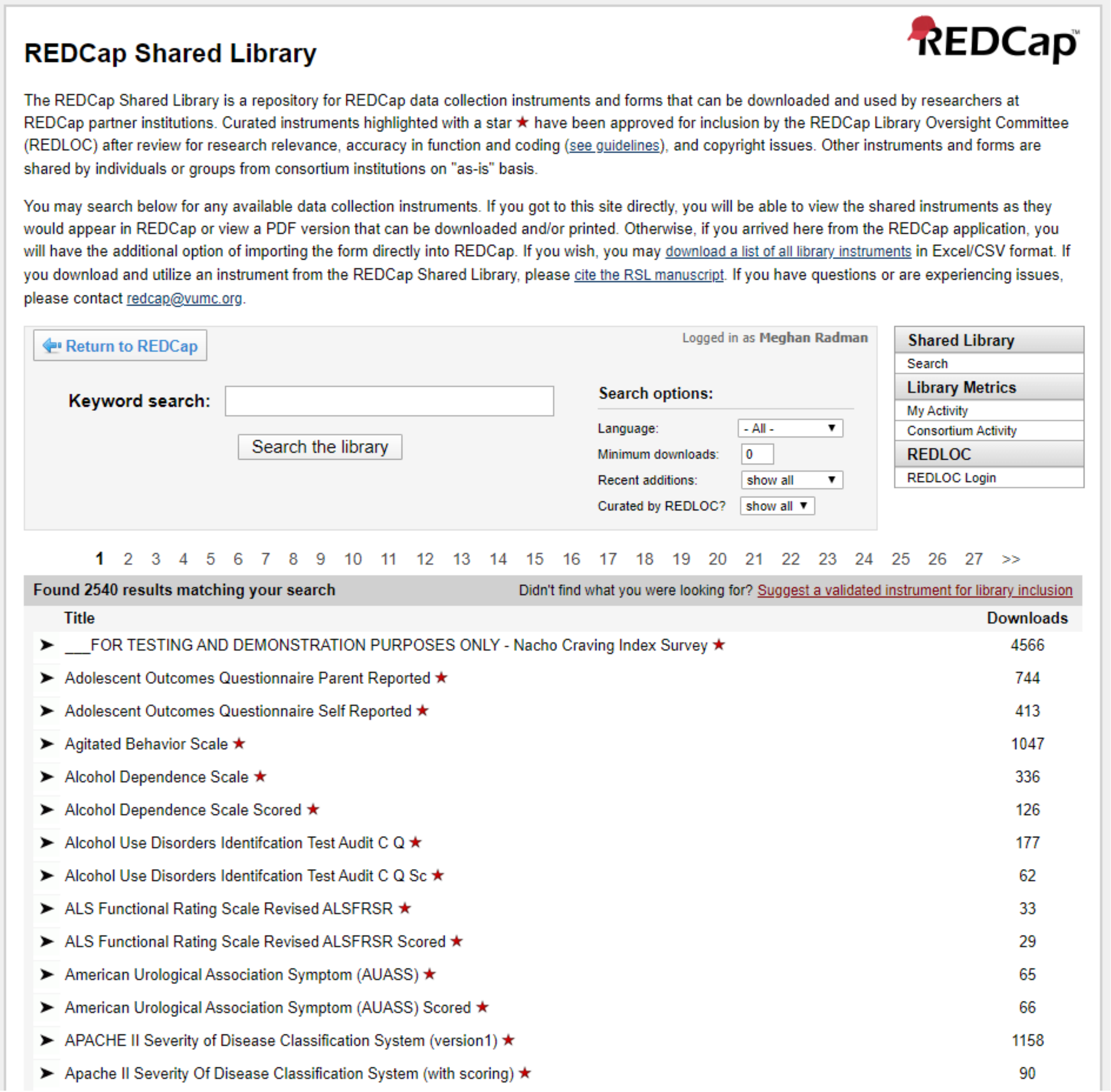

REDCap Shared Library

REDCap

The REDCap Shared Library is a repository for REDCap data collection instruments and forms that can be downloaded and used by researchers at REDCap partner institutions. Curated instruments highlighted with a star ★ have been approved for inclusion by the REDCap Library Oversight Committee (REDLOC) after review for research relevance, accuracy in function and coding (see guidelines), and copyright issues. Other instruments and forms are shared by individuals or groups from consortium institutions on "as-is" basis.

You may search below for any available data collection instruments. If you got to this site directly, you will be able to view the shared instruments as they would appear in REDCap or view a PDF version that can be downloaded and/or printed. Otherwise, if you arrived here from the REDCap application, you will have the additional option of importing the form directly into REDCap. If you wish, you may download a list of all library instruments in Excel/CSV format. If you download and utilize an instrument from the REDCap Shared Library, please cite the RSL manuscript. If you have questions or are experiencing issues, please contact redcap@vumc.org.

Return to REDCap

Logged in as **Meghan Radman**

Keyword search:

Search the library

Search options:

Language: - All -

Minimum downloads: 0

Recent additions: show all

Curated by REDLOC? show all

Shared Library
Search
Library Metrics
My Activity
Consortium Activity
REDLOC
REDLOC Login

1 2 3 4 5 6 7 8 9 10 11 12 13 14 15 16 17 18 19 20 21 22 23 24 25 26 27 >>

Found 2540 results matching your search Didn't find what you were looking for? Suggest a validated instrument for library inclusion

Title	Downloads
➤ ___FOR TESTING AND DEMONSTRATION PURPOSES ONLY - Nacho Craving Index Survey ★	4566
➤ Adolescent Outcomes Questionnaire Parent Reported ★	744
➤ Adolescent Outcomes Questionnaire Self Reported ★	413
➤ Agitated Behavior Scale ★	1047
➤ Alcohol Dependence Scale ★	336
➤ Alcohol Dependence Scale Scored ★	126
➤ Alcohol Use Disorders Identifcation Test Audit C Q ★	177
➤ Alcohol Use Disorders Identifcation Test Audit C Q Sc ★	62
➤ ALS Functional Rating Scale Revised ALSFRSR ★	33
➤ ALS Functional Rating Scale Revised ALSFRSR Scored ★	29
➤ American Urological Association Symptom (AUASS) ★	65
➤ American Urological Association Symptom (AUASS) Scored ★	66
➤ APACHE II Severity of Disease Classification System (version1) ★	1158
➤ Apache II Severity Of Disease Classification System (with scoring) ★	90

8. In the “Keyword search:” box, type “COMPASS” and press “Search the Library”

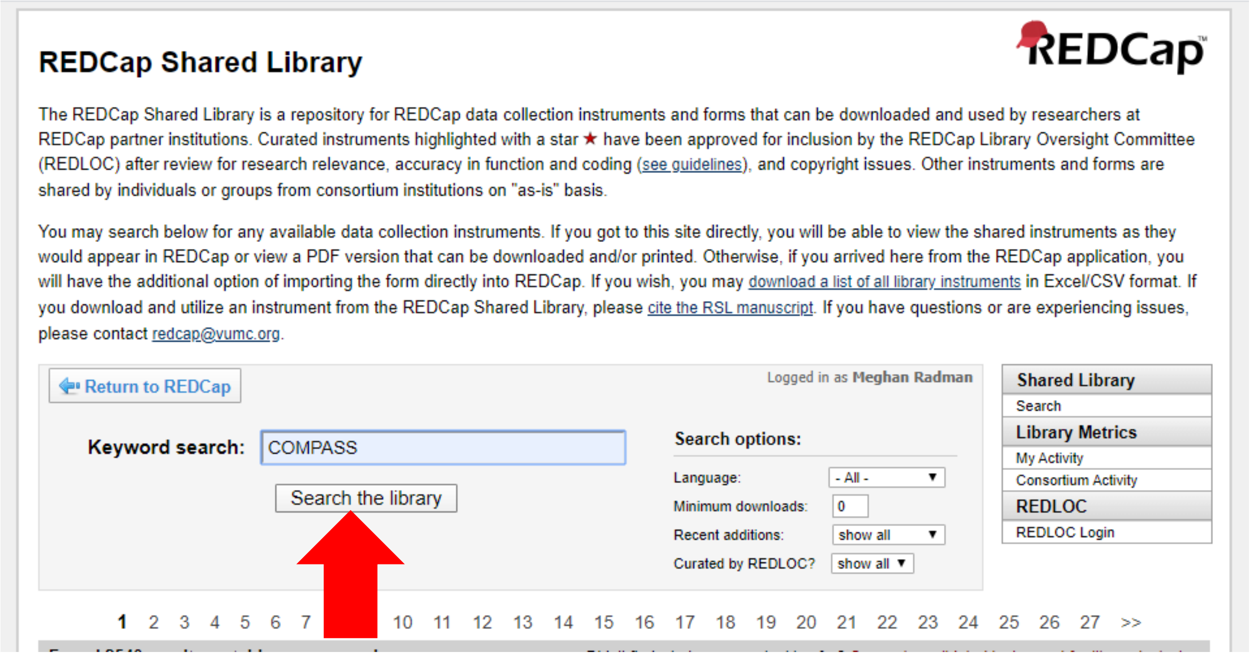

9. You will be shown a list of seven (7) instruments for the Engagement Tracker. You will need to import each instrument into your REDCap project separately. To do so, complete the steps below for each instrument

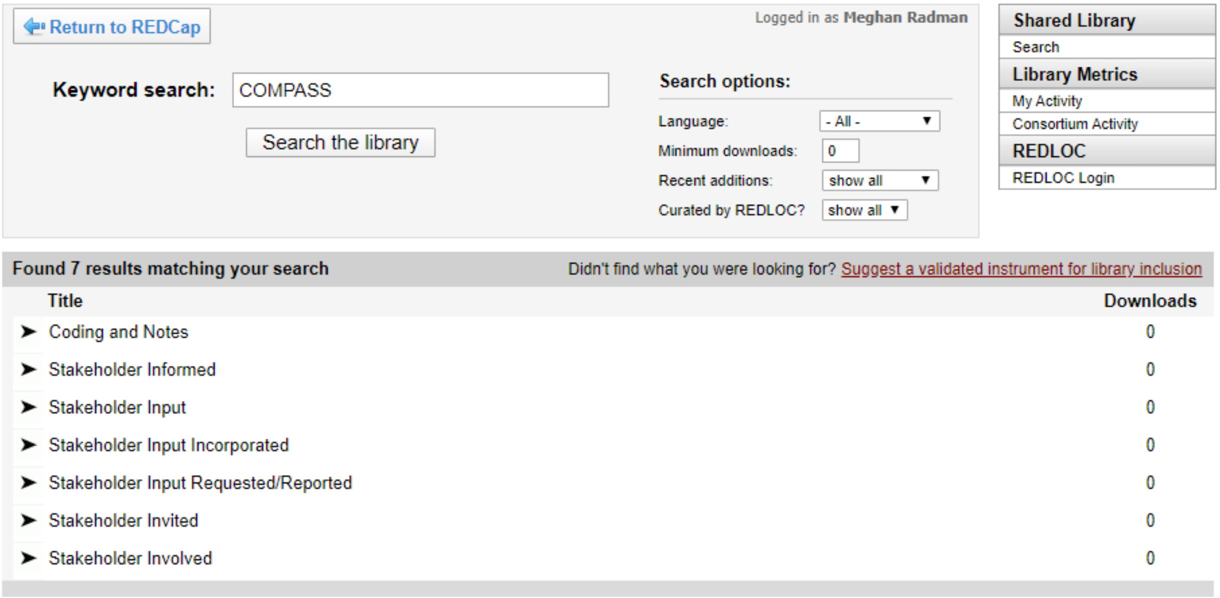

10. Click on the arrow ➤ to expand an instrument.

11. Click on the box “Import into my REDCap project”

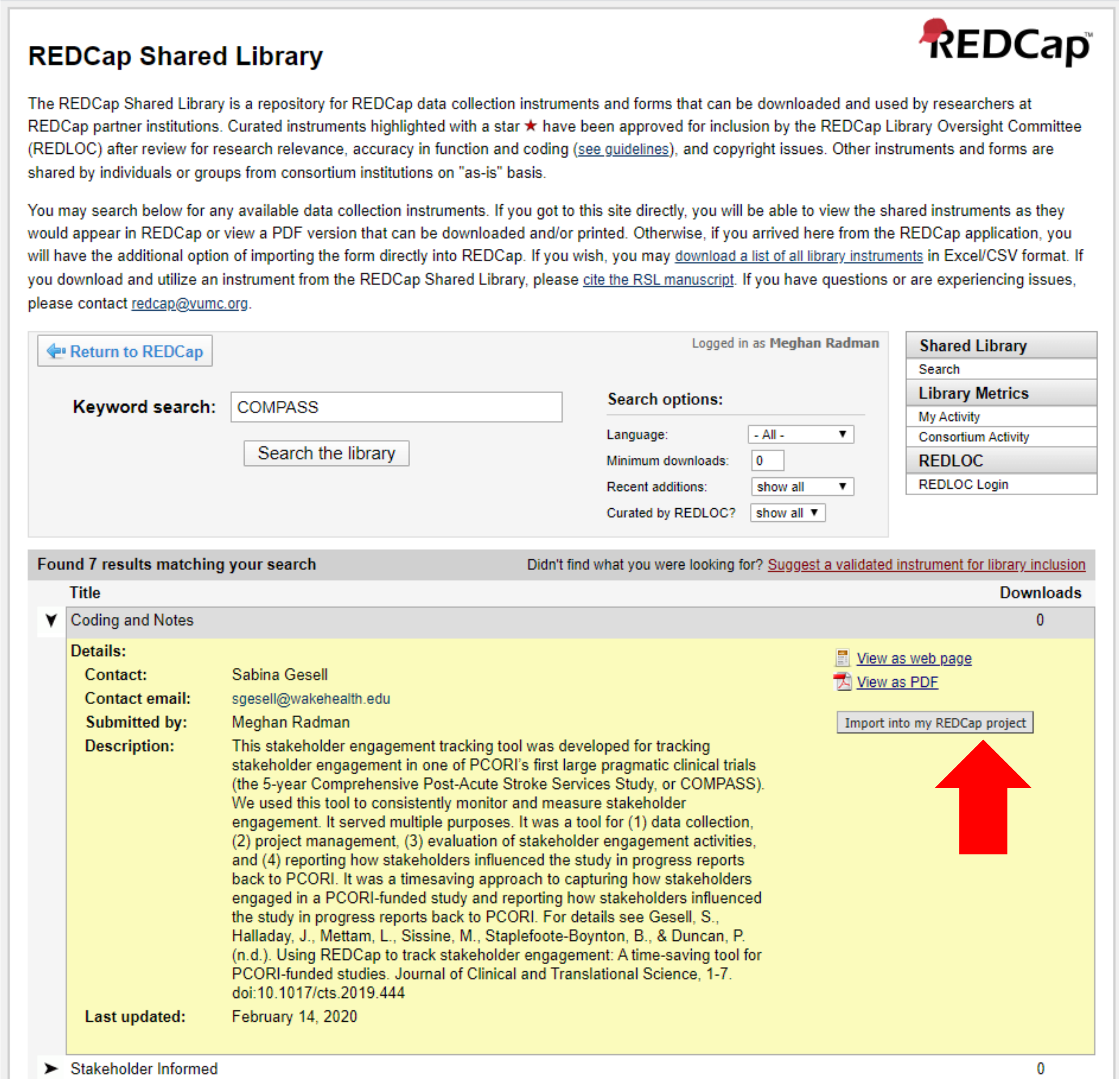

REDCap Shared Library

REDCap

The REDCap Shared Library is a repository for REDCap data collection instruments and forms that can be downloaded and used by researchers at REDCap partner institutions. Curated instruments highlighted with a star ★ have been approved for inclusion by the REDCap Library Oversight Committee (REDLOC) after review for research relevance, accuracy in function and coding (see guidelines), and copyright issues. Other instruments and forms are shared by individuals or groups from consortium institutions on "as-is" basis.

You may search below for any available data collection instruments. If you got to this site directly, you will be able to view the shared instruments as they would appear in REDCap or view a PDF version that can be downloaded and/or printed. Otherwise, if you arrived here from the REDCap application, you will have the additional option of importing the form directly into REDCap. If you wish, you may download a list of all library instruments in Excel/CSV format. If you download and utilize an instrument from the REDCap Shared Library, please cite the RSL manuscript. If you have questions or are experiencing issues, please contact redcap@vumc.org.

Return to REDCap

Logged in as **Meghan Radman**

Keyword search: COMPASS

Search the library

Search options:

Language: - All -

Minimum downloads: 0

Recent additions: show all

Curated by REDLOC? show all

Shared Library
Search
Library Metrics
My Activity
Consortium Activity
REDLOC
REDLOC Login

Found 7 results matching your search

Didn't find what you were looking for? Suggest a validated instrument for library inclusion

Title	Downloads
Coding and Notes	0

Details:

Contact: Sabina Gesell

Contact email: sgesell@wakehealth.edu

Submitted by: Meghan Radman

Description: This stakeholder engagement tracking tool was developed for tracking stakeholder engagement in one of PCORI's first large pragmatic clinical trials (the 5-year Comprehensive Post-Acute Stroke Services Study, or COMPASS). We used this tool to consistently monitor and measure stakeholder engagement. It served multiple purposes. It was a tool for (1) data collection, (2) project management, (3) evaluation of stakeholder engagement activities, and (4) reporting how stakeholders influenced the study in progress reports back to PCORI. It was a timesaving approach to capturing how stakeholders engaged in a PCORI-funded study and reporting how stakeholders influenced the study in progress reports back to PCORI. For details see Gesell, S., Halladay, J., Mettam, L., Sissine, M., Staplefoote-Boynton, B., & Duncan, P. (n.d.). Using REDCap to track stakeholder engagement: A time-saving tool for PCORI-funded studies. Journal of Clinical and Translational Science, 1-7. doi:10.1017/cts.2019.444

Last updated: February 14, 2020

View as web page

View as PDF

Import into my REDCap project

Title	Downloads
Stakeholder Informed	0

12. Read through the “Shared Content Agreement” and click “I agree”

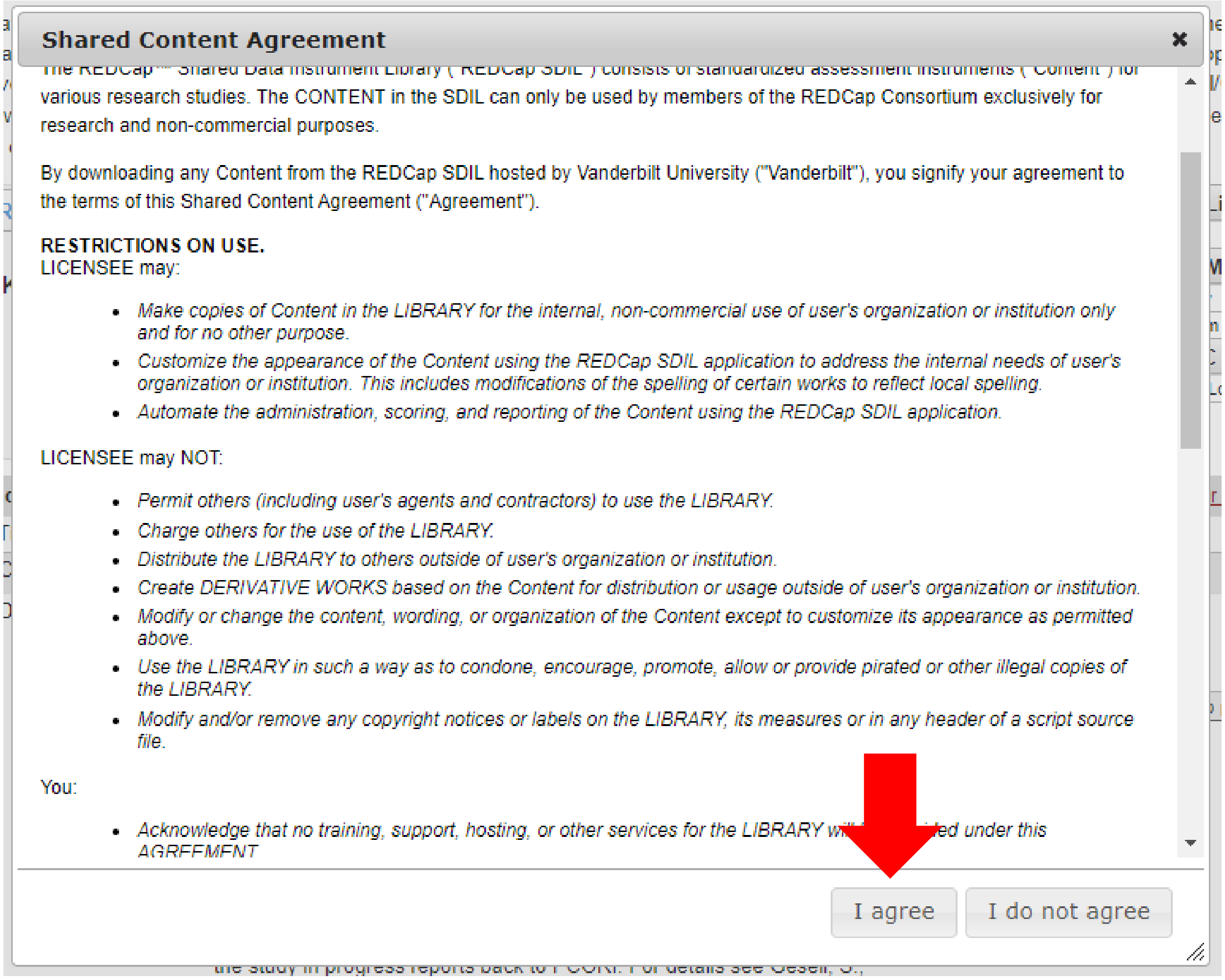

13. You will then be taken back into your REDCap Project. Click “Add” to start the import process.

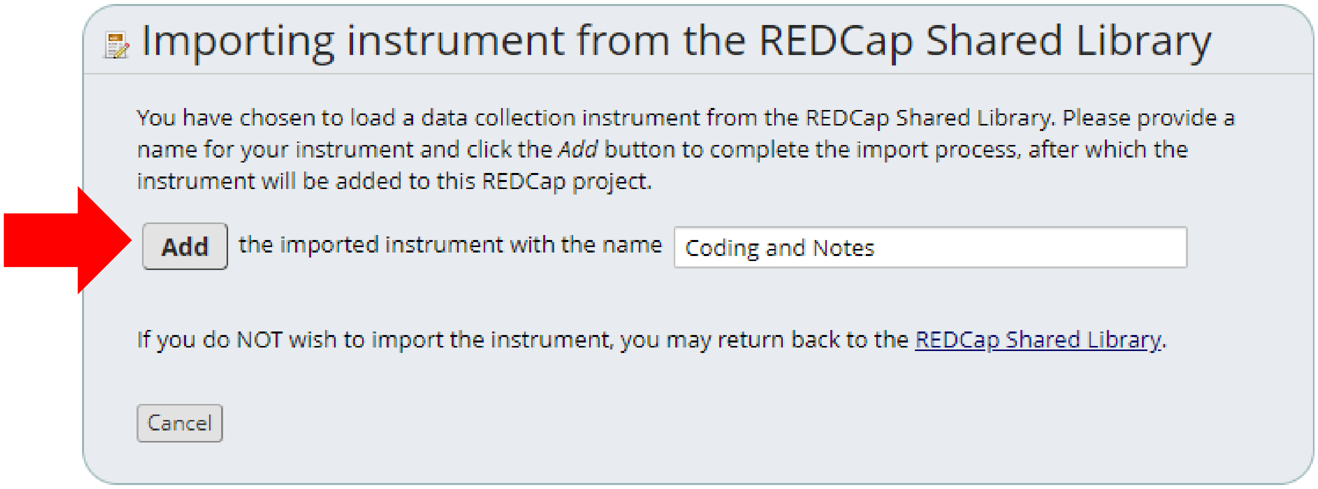

14. Repeat steps five (5) through thirteen (13) to add all seven (7) instrument in your project.
15. The order of the instruments in your project should be:
    1. Stakeholder Input Requested/Reported
    2. Stakeholder Invited
    3. Stakeholder Input
    4. Stakeholder Involved
    5. Stakeholder Input Incorporated
    6. Stakeholder Informed
    7. Coding and Notes
