# Supplementary material for: Using REDCap to track stakeholder engagement: A time-saving tool for PCORI-funded studies
Source: J Clin Transl Sci. 2020 Feb 6;4(2):108–14. doi: 10.1017/cts.2019.444 (PMC7159814; doi:10.1017/cts.2019.444)
Supplement: Supplementary file 1 [file S2059866119004448sup.zip › S2059866119004448sup002.pdf]

Stakeholder Engagement Activity Form

Please complete the survey below.

Thank you!

You are completing this form to:

◯ Report on a past engagement activity
◯ Request stakeholder input

Today's Date: ______________________

Name of person completing form: ______________________

Purpose of Engagement Activity: ______________________

Deadline for stakeholder feedback: ______________________

Stakeholder Invited

Please complete the survey below.

Thank you!

What method of engagement was or will be use?

- ☐ Scheduled presentation
- ☐ Stakeholder workshops
- ☐ Focus groups
- ☐ Stroke support group meetings
- ☐ Conference calls
- ☐ Video conference calls
- ☐ Webinars
- ☐ Joining already scheduled statewide and local meetings
- ☐ E-mail exchanges
- ☐ 1:1 phone calls
- ☐ In person meeting
- ☐ Embed Stakeholder in Committee/Working Group
- ☐ Other

What is the title of the presentation? ______________________________

What type of presentation is this? (check all that apply)

- ☐ Oral
- ☐ Poster
- ☐ Peer Reviewed

Please describe other method of engagement: ______________________________

Has this engagement activity been approved?

- ◯ Yes
- ◯ No

Invitation sent to stakeholder?

- ◯ Yes
- ◯ No
- ◯ N/A

Notes on inviting stakeholders for engagement:

____
(Please note any challenges or other special notes when reaching out to stakeholders for engagement)

Stakeholder Involved

Please complete the survey below to tell us more about the your engagement activity that. It should take about 10-15 minutes to complete.

Thank you!

Date of event or engagement activity: __________________________________

Name of stakeholder group(s) or individual engaged: __________________________________

Name of event or engagement activity: __________________________________

Who was engaged?

- ☐ Patients
- ☐ Family Caregivers
- ☐ Clinicians- Advance Practice Practitioners
- ☐ Clinicians- Stroke Care Coordinator / Post Acute Care Coordinators (RN)
- ☐ Clinicians- Primary Care Physicians
- ☐ Clinicians- Hospital-based Physicians
- ☐ Clinicians- Rehabilitation Therapists (PT, OT, Speech)
- ☐ Clinicians- Pharmacists
- ☐ Clinicians- Home health providers
- ☐ Clinic/ Hospital / Health System Representative
- ☐ Community-based Services Representative
- ☐ Patient/ Consumer/ Caregiver/ Advocacy Organization Representative
- ☐ Payer
- ☐ Purchaser
- ☐ Policy Maker
- ☐ Industry
- ☐ Other

Please describe other stakeholder involved: __________________________________

Describe the event and/or the purpose of the engagement activity: ____

Location of event or engagement activity:
(When providing the location, clarify if you went to the stakeholder or they came to you) ____

Please see below for definitions of the different levels for engagement. Use this to determine the Engagement Level for this engagement activity in the next questions.

Engagement Level	Defintion	Example
1. Information Sharing	Simply informing patients or stakeholders about resarch activities	Communicating plans to the patient community
2. Consulation	Consulting patients or stakeholders on decisions	Offering opinions, advice, feedback
3. Collaboration	Deciding together with stakeholders; Acting together with stakeholders	Joint decision solicited; Taking actions jointly
4. Patient/stakeholder direction	Encouraging independent initiatives by patienst and stakeholders	Actions that can lead to patient/caregiver/organiztion generated research

Please describe the primary level of engagement involved in this activity?

- ◯ Information Sharing
- ◯ Consultation
- ◯ Collaboration
- ◯ Patient/stakeholder direction

Please describe the secondary level of engagement involved in this activity?

- ◯ Information Sharing
- ◯ Consultation
- ◯ Collaboration
- ◯ Patient/stakeholder direction

Presenter/Leader(s): ______________________________

Did a stakeholder co-present (Y/N)?

- ◯ Yes
- ◯ No
- ◯ N/A

Estimated number in attendance: ______________________________

Phase of study:

- ◯ Planning/Design
- ◯ Conducting and Monitoring
- ◯ Dissemination and Implementation

Describe stakeholder input on study:
(Please indicate if this input has been incorporated or is being evaluated. Type in N/A if not applicable.) ______________________________

Did you face any challenges with engaging these stakeholders?

- ◯ Yes
- ◯ No

Describe any challenges you faced engaging these stakeholders. ______________________________

Were you able to resolve these challenges?

- ◯ Yes
- ◯ No
- ◯ Partially

What were your creative solutions?

____

Stakeholder Input Incorporated

Has input been vetted by representatives of the Executive Committee?

- ◯ Yes
- ◯ No
- ◯ N/A

Was stakeholder input incorporated in the study?

- ◯ Yes
- ◯ No
- ◯ N/A

Describe impact of stakeholder input on study:

____

Why was input NOT included?

____

Social Ecological Model Representation:

(To be filled out by Engagement Team)

- ☐ Public Policy
- ☐ Community
- ☐ Institutional
- ☐ Social Network
- ☐ Individual

Stakeholder Informed

Did you or a COMPASS representative follow-up with stakeholders?

◯ Yes
◯ No
◯ N/A

Would you like someone from the Engagement team to follow-up? If so, please tell us how.

____

What method(s) was used to follow-up with stakeholders?

☐ Stakeholder workshops
☐ Focus groups
☐ Stroke support group meetings
☐ Conference calls
☐ Video conference calls
☐ Webinars
☐ Joining already scheduled statewide and local meetings
☐ E-mail exchanges
☐ 1:1 phone calls
☐ In person meetings at location convenient for stakeholder
☐ Other

Please describe other method for informing the stakeholder:

____

Notes about how we followed-up with stakeholders:

____

Coding and Notes

This entry is a duplicate
◯ Yes
◯ No

Roadmap for stakeholder engagement in the COMPASS Study.

STUDY OVERSIGHT ACTIVITIES

☐ Participate in Steering Committee meetings
☐ Participate in Core Engagement Committee to monitor engagement process

INTERVENTION ACTIVITIES

☐ Design intervention - Caregiver
☐ Design intervention - Recovery & Physical Activity
☐ Design intervention - Secondary Prevention & Medication Management
☐ Design intervention - Community Resources
☐ Design intervention - Transitional Care Survey
☐ Develop messaging and marketing to patients and providers
☐ Define goals / screener questions for 2 day call
☐ Define goals / measures for 7-14 day clinic - Functional Assessment
☐ Define goals / measures for 7-14 day clinic - Medical and Neurological Assessment
☐ Define goals / measures for 7-14 day clinic - E-Care Plan and Database
☐ Define goals / measures for 7-14 day clinic - Referrals
☐ Define goals / measures for 7-14 day clinic - Caregiver Assessment
☐ Define goals for 30- & 60-day follow-up calls
☐ Identify community resources to support recovery after discharge
☐ Training of hospital- and community-based clinicians
☐ Job description for PAC
☐ Study website - input on website content, user friendliness

OUTCOME MEASUREMENT ACTIVITIES

☐ Finalize 90 day patient outcome measurement
☐ Finalize 95 -110 day caregiver survey
☐ Refine data collection forms (including enrollment form)
☐ Refine telephone scripts used with patient at data collection
☐ Consent process
☐ Methods of data collection, dealing with non-response
☐ Useful ways to engage patients/caregivers between discharge and 90 day data collection
☐ Input on claims-based outcome measures (mortality, hospitalization, physician visits, medication use, etc.)

QI IMPROVEMENTS ACTIVITIES

☐ Guide development and reporting of the QI metrics most meaningful for providers and healthcare systems

RECRUITMENT & RETENTION OF HOSPITALS AND PATIENTS ACTIVITIES

☐ Informational brochure on quality improvement initiative
☐ Incentives to follow stroke patients and collect relevant data in a timely manner
☐ Input on best ways to identify and follow stroke patients and determine study-eligible patients
☐ Input on ways to keep hospital/staff engaged in study
☐ Methods for monitoring and maintaining completeness of patient enrollment
☐ Incentives for hospitals to fully engage in trial

DATA ANALYSIS ACTIVITIES

- ☐ Formulate secondary questions that matter to stakeholders

DISSEMINATION ACTIVITIES

- ☐ Refine D&I plan
- ☐ Leverage stakeholders' networks to maximize reach
- ☐ Disseminate study information and final results across the state
- ☐ Identify barriers for dissemination
- ☐ Identify most effective dissemination strategies to ensure timely and effective communication to patients, community leaders, hospital administrators, policy makers
- ☐ Educate local (county commissioners) and state policy makers (Senators) to understand kind of research we are doing, why it is important, why NC is uniquely positioned to do this and become a national leader, understand barriers to providers and patients
